# Intraluminal Apple as a Rare Cause of Small Bowel Obstruction

**DOI:** 10.1155/2018/1304519

**Published:** 2018-12-13

**Authors:** Koichi Inukai, Akihiro Usui, Motohiko Yamada, Koji Amano, Nobutaka Mukai, Yusuke Tsunetoshi, Yasuki Nakata, Junichiro Yokota

**Affiliations:** Department of Acute Care Surgery, Sakai City Medical Center, 1-1-1 Ebaraji-cho, Nishi-ku, Sakai, Osaka 593-8304, Japan

## Abstract

Small bowel obstruction due to ingested foreign bodies is rare in adults. A 48-year-old male visited our hospital with abdominal pain and vomiting. Computed tomography revealed intestinal obstruction by a 3 × 4 cm apple-shaped foreign body. Emergency surgery was performed to clear the obstruction which, upon inspection, was caused by a sexual toy made of rubber. Flexible rubber products that are ingested should be carefully followed after they pass thorough the pylorus. For obstructions related to sexual behavior, the patient's sense of shame often delays the process of seeking medical attention, thereby making preoperative diagnosis difficult.

## 1. Introduction

Bowel obstruction is defined by the hindrance to the progression of the intestinal content due to a mechanical obstacle. Small bowel obstruction in a virgin abdomen is rare; however, the main causes of such obstructions are hernias, malignancies, and inflammatory bowel disease. Ingested foreign bodies are particularly rare in adults and can obstruct the intraluminal small bowel. Observed obstructions often include food items, plastics, metals, plants, soil, hair, and insects [1, 2]. Obtaining a comprehension history of a patient's diet as well as clinical imaging renders preoperative diagnosis of ingested foreign bodies possible. The objectives of imaging in a patient with clinically suspected intestinal obstruction are (1) to confirm that it is a true obstruction and to differentiate it from a condition of spastic ileus; (2) to define the level and the cause of the obstruction; (3) to search for findings of strangulation; (4) to allow a correct management (either medically or surgically by laparotomy or laparoscopy). Herein, we report a unique case of a small bowel obstruction caused by a foreign body.

## 2. Case Presentation

**Figure 1:**
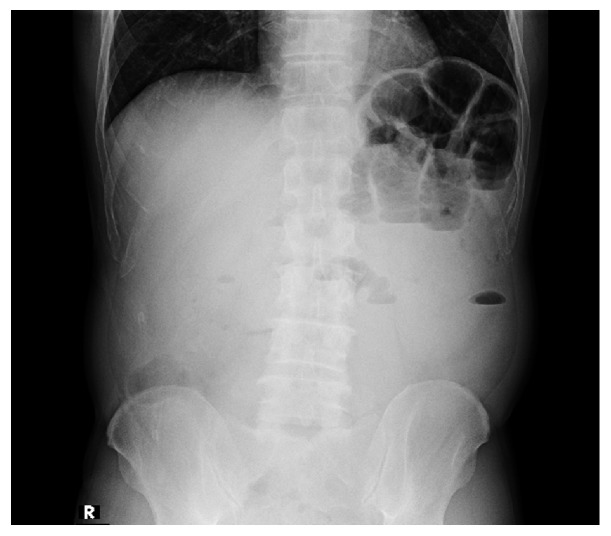
Abdominal radiography performed in upright position revealed distended loops of small bowel containing gas and fluid in the left upper abdomen.

**Figure 2:**
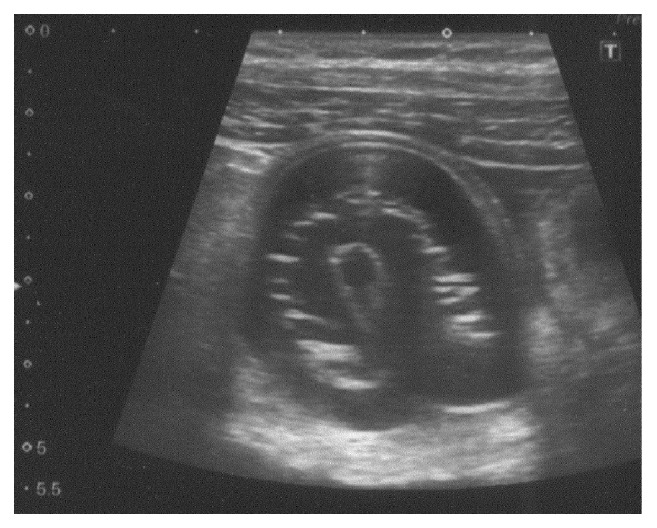
Abdominal ultrasonography revealed an apple-shaped foreign body.

**Figure 3:**
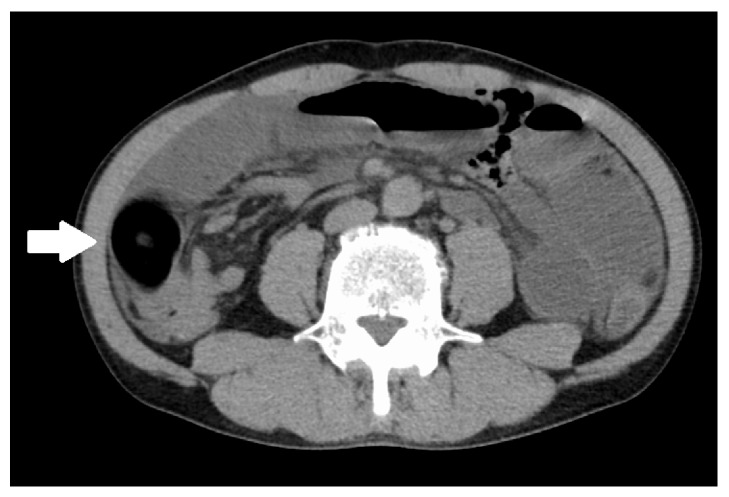
Computed tomography revealed intestinal obstruction by a 3 × 4 cm foreign body (white arrow) within the right lower abdominal cavity.

A 48-year-old male presented to our hospital with abdominal pain and vomiting. He had no history of prior surgery. His physical examination indicated a body temperature of 37.1°C, blood pressure of 136/61 mmHg, and pulse rate of 94 bpm. He had slight tenderness to palpation over the entire abdomen. His laboratory findings were only significant for an abnormally elevated white blood cell count (14,400/mm^3^). Abdominal radiography performed in upright position revealed distended loops of small bowel containing gas and fluid in the left upper abdomen and absence of pneumoperitoneum (Figure 1). Abdominal ultrasonography revealed an apple-shaped foreign body (Figure 2). Computed tomography was performed without intravenous contrast medium administration. It revealed intestinal obstruction by a 3 × 4 cm foreign body within the right lower abdominal cavity, with dilatation of the small intestine at the proximal side (Figure 3). Based on this clinical picture, the patient was diagnosed as having small bowel obstruction secondary to a foreign body. An emergency surgery was thus performed.

**Figure 4:**
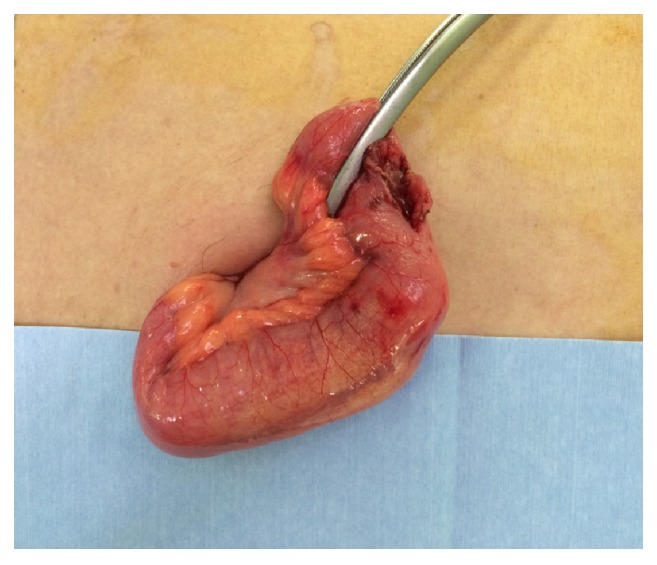
The abnormally dilated small bowel is exteriorized. A luminal obstructing foreign body was palpable.

**Figure 5:**
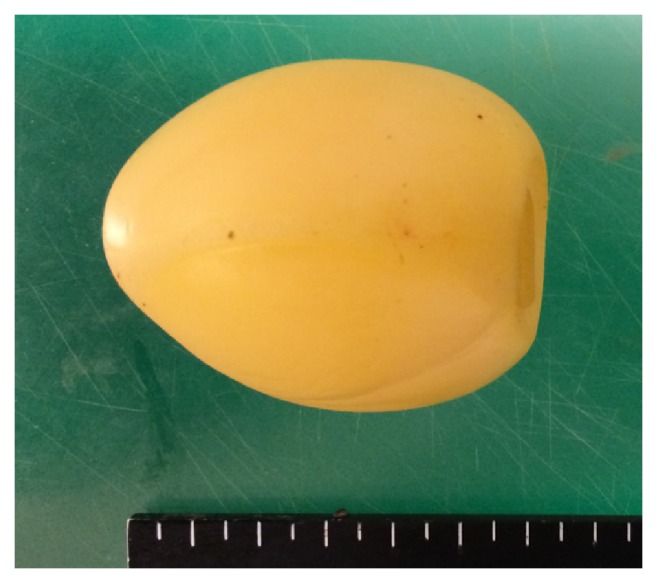
The foreign body was a sex toy made with soft rubber. The original size was 5.5 × 6.5cm.

We performed a 4-cm small abdominal incision by tracing the small intestine from the terminal ileum; further, the site of foreign body was identified by locating the point 40 cm from the proximal side of the terminal ileum. Thereafter, the foreign body was extracted using enterotomy (Figure 4). The foreign body was a sexual toy designed to attach to the glans penis for the purpose of masturbation (Figure 5). The toy, having been made of rubber, was extremely soft and compactly foldable. Hence, when swallowed, the toy entered the small intestine and caused an obstruction at the ileum.

The patient experienced surgical site infection after surgery; however, conservative therapy improved his condition within a short period of time. The patient was discharged from the hospital 10 days after the surgery.

## 3. Discussion

Intraluminal foreign bodies can manifest secondary to swallowing [3, 4] or via rectal insertion [5, 6]. Abdominal foreign body related complications include intestinal obstruction, gastrointestinal tract perforations, peritonitis, and hepatic abscesses [7, 8]. Once the object passes through the pylorus, it usually spontaneously reaches the rectum without morbidity.

In our case, the rubber foreign body could not reach the rectum because of its size. Rubber products can change their shape to some extent; hence, even when the object passes through the pylorus, its changing shape may prevent it from moving through the distal intestinal tract. Soft and flexible objects therefore warrant close and continual observation after passing through the pylorus.

The incidence of rectal foreign bodies has recently increased, and many such cases involve a bottle or glass [9]. Most cases of anal foreign body insertions are associated with sexual behaviors [10]. Some patients may wait prior to visiting a hospital because they feel ashamed and do not want to disclose information regarding their private lives. In our case, the patient denied swallowing any unusual foreign body; hence, we were unable to preoperatively identify the foreign body. However, when we questioned the patient postoperatively, he admitted to accidentally swallowing the toy while being under the influence of alcohol. Small bowel obstructions caused by sex toys are extremely rare and are therefore difficult to diagnose. Consequently, close attention should be paid to such patients.

## References

[1] Zamora I. J., Vu L. T., Larimer E. L., Olutoye O. O. (2012). Water-absorbing balls: A "growing" problem. *Pediatrics*.

[2] Ooi S., Hong K. (2015). Small bowel obstruction caused by dried apple. *International Journal of Surgery Case Reports*.

[3] Gümüs M., Kapan M., Önder A., Tekbas G., Yagmur Y. (2011). An unusual cause of small bowel obstruction: Dried apricots. *Journal of the Pakistan Medical Association*.

[4] Poynter B. A., Hunter J. J., Coverdale J. H., Kempinsky C. A. (2011). Hard to swallow: A systematic review of deliberate foreign body ingestion. *General Hospital Psychiatry*.

[5] Pinto A., Miele V., Pinto F. (2015). Rectal Foreign Bodies: Imaging Assessment and Medicolegal Aspects. *Seminars in Ultrasound, CT and MRI*.

[6] Coskun A., Erkan N., Yakan S., Yldirim M., Cengiz F. (2013). Management of rectal foreign bodies. *World Journal of Emergency Surgery*.

[7] Laterre P.-F., Dangoisse C. (2014). Tracking the foreign body, a rare cause of hepatic abscess. *BMC Gastroenterology*.

[8] Litovitz T., Schmitz B. F. (1992). Ingestion of cylindrical and button batteries: an analysis of 2382 cases. *Pediatrics*.

[9] Lake J. P., Essani R., Petrone P., Kaiser A. M., Asensio J., Beart R. W. (2004). Management of retained colorectal foreign bodies: predictors of operative intervention. *Diseases of the Colon & Rectum*.

[10] Anderson K. L., Dean A. J. (2011). Foreign bodies in the gastrointestinal tract and anorectal emergencies. *Emergency Medicine Clinics of North America*.

